# A randomized controlled trial of conventional GVHD prophylaxis with or without teprenone for the prevention of severe acute GVHD

**DOI:** 10.1007/s00277-025-06269-2

**Published:** 2025-02-24

**Authors:** Wataru Kitamura, Keiko Fujii, Mitsuru Tsuge, Toshiharu Mitsuhashi, Hiroki Kobayashi, Chihiro Kamoi, Akira Yamamoto, Takumi Kondo, Keisuke Seike, Hideaki Fujiwara, Noboru Asada, Daisuke Ennishi, Ken-ichi Matsuoka, Nobuharu Fujii, Yoshinobu Maeda

**Affiliations:** 1 Department of Hematology and Oncology, Okayama University Hospital, 2-5-1 Shikata-Cho, Kita-Ku, Okayama, 700-8558 Japan; 2 Division of Transfusion and Cell Therapy, Okayama University Hospital, Okayama, Japan; 3 Division of Clinical Laboratory, Okayama University Hospital, Okayama, Japan; 4 Department of Pediatric Acute Diseases, Okayama University Academic Field of Medicine Dentistry, and Pharmaceutical Sciences, Okayama, Japan; 5 Center for Innovative Clinical Medicine, Okayama University Hospital, Okayama, Japan; 6 Center for Comprehensive Genomic Medicine, Okayama University Hospital, Okayama, Japan

**Keywords:** Allogeneic hematopoietic stem cell transplantation, Graft-versus-host disease, Teprenone, Oxidative stress, Interleukin-33

## Abstract

Therapies that effectively suppress graft-versus-host disease (GVHD) without compromising graft-versus-leukemia/lymphoma (GVL) effects is important in allogeneic hematopoietic stem cell transplantation (allo-HSCT) for hematopoietic malignancies. Geranylgeranylacetone (GGA) is a main component of teprenone, a gastric mucosal protectant commonly used in clinical practice. In preclinical models, GGA suppresses proinflammatory cytokines, including interleukin (IL)-1β, IL-6, and tumor necrosis factor-α (TNF-α), which are associated with GVHD as well as induces thioredoxin-1 (Trx-1), which suppresses GVHD while maintaining GVL effects. Here, we investigated whether the addition of teprenone to standard GVHD prophylaxis could reduce the cumulative incidence of severe acute GVHD (aGVHD) without attenuating GVL effects. This open-label, randomized clinical trial enrolled 40 patients (21 control and 19 teprenone group) who received allo-HSCT between May 2022 and February 2023 in our institution. Patients in the teprenone group received 50 mg of teprenone orally thrice daily for 21 days from the initiation of the conditioning regimen. The cumulative incidence of severe aGVHD by day 100 after allo-HSCT was not significantly different in the two groups (27.9 vs. 16.1%, *p* = 0.25). The exploratory studies revealed no obvious changes in Trx-1 levels, but the alternations from baseline in IL-1β and TNF-α levels at day 28 after allo-HSCT tended to be lower in the teprenone group. In conclusion, we could not demonstrate that teprenone significantly prevented the development of severe aGVHD. Discrepancy with preclinical model suggests that appropriate dose of teprenone may be necessary to induce the expression of antioxidant enzymes that suppress severe aGVHD. Clinical Trial Registration number:jRCTs 061210072.

**Supplementary information:**

The online version contains supplementary material available at 10.1007/s00277-025-06269-2.

## Introduction

Allogeneic hematopoietic stem cell transplantation (allo-HSCT) has been established as a potential curative treatment for various hematopoietic tumors [1]. The therapeutic principle of the allogeneic immune response provides graft-versus-leukemia/lymphoma (GVL) effects but also has two sides: GVL and graft-versus-host disease (GVHD) [2]. Most of the approved therapeutic strategies for GVHD relyed on immunosuppressive therapy and enhanced GVHD prophylaxis may lead to an increased risk of relapse due to a reduction in GVL effects. Therefore, it is important to identify novel therapeutic strategies to maintain the efficacy of GVL [3]. There have been recently reported that the addition of novel agents focused on immunomodulatory such as cytotoxic T-cell-lymphocyte-4-immunoglobulin, dipeptidyl peptidase 4 inhibitor, and gut-selective anti-α_4_β_7_ integrin monoclonal antibody to conventionl GVHD prophylaxis may help reduce the incidence of severe acute GVHD (aGVHD) after allo-HSCT and improve patient outcomes [4–6].

Based on the transition of therapeutic strategies for GVHD described above, we focused on strategy to inhibit oxidative stress associated with T cell activation and cytokine dysregulation [7, 8]. Oxidative stress is an inevitable consequence of allo-HSCT and an important factor in exacerbating GVHD [9]. It is elevated in allo-HSCT recipients because the conditioning regimen prior to allo-HSCT increases cellular reactive oxygen species (ROS) levels [10, 11]. Heat shock protein (HSP)−70 and thioredoxin-1 (Trx-1), antioxidant enzymes with cytoprotective effects against ROS, are induced by geranylgeranylacetone (GGA), the main component of teprenone, a commonly used gastric mucosal protectant in Japanese daily clinical practice [12, 13]. Increased expression and genetic polymorphisms of HSP-70 correlate with GVHD [14, 15]. Increased Trx-1 levels reduce the sensitivity of human regulatory T cells to oxidative stress, contributing to the prevention of prolonged or excessive immune responses in vitro [16]. Trx-1 administration suppressed GVHD in a murine model by reducing proliferation, migration to the GVHD target organs, interferon (IFN)-γ production, and ROS accumulation of donor T cells, while maintaining GVL effects [17]. Moreover, although the reports did not explore the association with GVHD, GGA suppresses proinflammatory cytokines associated with GVHD, such as interleukin (IL)-1β, IL-6, and tumor necrosis factor-α (TNF-α) [18–21].

Teprenone, which is mainly composed of GGA, has been approved in Asia as a safe gastric mucosal protectant that exerts its cytoprotective effects by inducing HSP and Trx-1 [22]. Based on previous preclinical studies [17–19], we hypothesized that teprenone (i.e., GGA) administration in the peri-allo-HSCT period may reduce the incidence of severe aGVHD via the induction of Trx-1 and/or suppression of inflammatory cytokines associated with GVHD, while maintaining the GVL effects. In this trial, we aimed to investigate the efficacy and safety of teprenone in combination with conventional GVHD prophylaxis to reduce the incidence of severe aGVHD.

## Methods

### Study design

This open-label, randomized trial evaluated the efficacy and safety of teprenone in combination with conventional GVHD prophylaxis for severe aGVHD after allo-HSCT at our institution. The protocol was approved by Okayama University (CRB21-010) and published in the Japan Registry of Clinical Trials (jRCTs061210072) on January 27, 2022. This study was conducted following compliance with the principles of the 1964 Declaration of Helsinki and its later amendments. Informed consent was obtained from all patients prior to any screening procedure or inclusion.

We randomized 40 patients hospitalized for allo-HSCT into the teprenone and no-teprenone groups in a 1:1 ratio using an online randomization system (the Internet Data and Information System for Clinical and Epidemiological Research, a cloud version of the University Medical Information Network).

### Patient selection

The inclusion criteria were as follows: (i) age ≥ 15 years at the time of enrolment, (ii) provision of written informed consent, and (iii) no conflicts with the exclusion criteria. The exclusion criteria were as follows: (i) uncontrolled psychiatric symptoms; (ii) pregnancy, lactation, or possibly pregnancy; and (iii) history of serious allergy to teprenone. To minimize bias, consecutive patients referred to our institution between May 2022 and February 2023 were included in this study.

### GVHD prophylaxis and intervention

The prophylaxis of aGVHD in patients with matched unrelated donor (MUD) bone marrow (BM), or matched sibling donor (MSD) or MUD peripheral blood stem cells (PBSCs) as a stem cell source was tacrolimus (TAC) (0.02 mg/kg from day − 1, with the dose being modified to target serum trough levels of 10–12 ng/mL) + methotrexate (10–7-7 mg/m^2^ on days + 1, + 3, and + 6). In addition, patients whose stem cell source was 1-locus mismatch unrelated donor (MMUD) BM received rabbit antithymocyte globulin (1.25–2.5 mg/kg on day − 4 and/or − 3) at the discretion of the attending physician. The prophylaxis for aGVHD in patients with cord blood as a stem cell source was TAC (dose as described above) + mycophenolate mofetil (MMF) (30 mg/kg/day with a maximum dose of 2000 mg/day from day − 1). The prophylaxis of aGVHD in patients with haploidentical PBSCs as the stem cell source was post-transplant cyclophosphamide (PTCy) (40–50 mg/kg on days + 3 and + 4) in combination with TAC + MMF (both dose as described above).

**Fig. 1:**
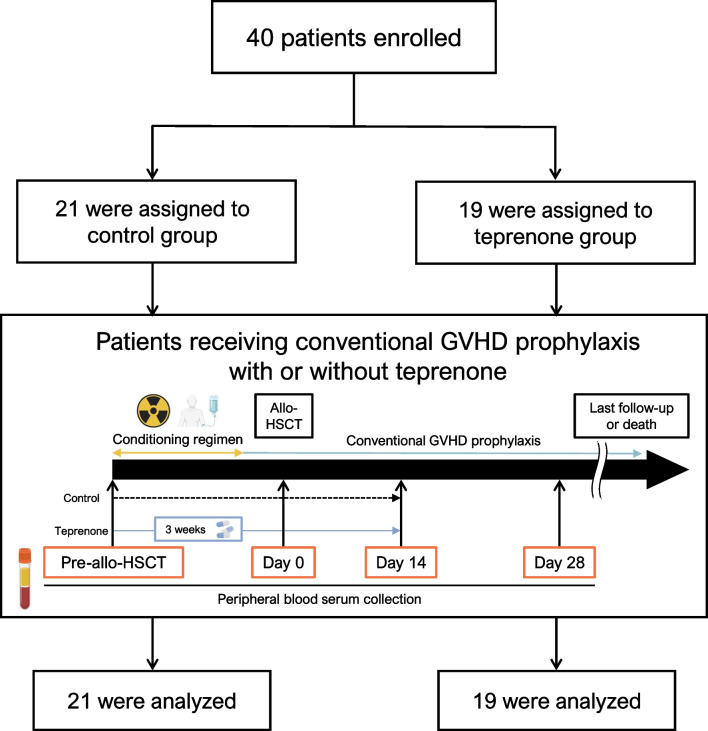
A flowchart of patient selection and the study design. The schema was partially created using BioRender.com. Abbreviation: allo-HSCT, allogeneic hematopoietic stem cell transplantation; GVHD, graft-versus-host disease

Patients in the teprenone group received 50 mg of teprenone orally thrice a day (the dose approved by the insurance in Japan) for 21 days from the initiation of the conditioning regimen unless they developed intolerable toxicities (Fig. 1). With regard to the duration of teprenone, we decided on the basis of previous reports (oxidative stress was increased after initiation of the conditioning regimen in allo-HSCT recipients and administration of recombinant Trx-1 for 2 weeks post-transplantation significantly reduced proliferaton, ROS accumulation, and IFN-γ production of donor T cells in murin model [10, 11, 17]). We imposed no limitations on other treatments during allo-HSCT that the attending physicians deemed necessary, but the use of drugs with overlapping GGA components was prohibited.

### Endpoints and definitions

The primary endpoint was the cumulative incidence of severe aGVHD (grade ≥ III) by day 100 after allo-HSCT. The secondary endpoint was overall survival (OS). Despite not being a prespecified endpoint in the protocol, the cumulative incidence of non-relapse mortality (NRM), relapse, and moderate-to-severe chronic GVHD (cGVHD) was estimated.

Neutrophil engraftment was defined as the first day of 3 consecutive days with absolute neutrophil counts ≥ 0.5 × 10^9^/L. Platelet engraftment was defined as the first day of 7 consecutive days with absolute platelet counts ≥ 20 × 10^9^/L without platelet transfusion. aGVHD and cGVHD were diagnosed and graded based on the traditional criteria [23, 24]. OS was defined as the time from allo-HSCT to death from any cause or until the last follow-up date for patients who were alive. Performance status (PS) was graded according to the Eastern Cooperative Oncology Group (ECOG) guidelines [25]. The hematopoietic cell transplantation-specific comorbidity index (HCT-CI) was calculated according to a previous report [26]. The disease risk was determined using the refined disease risk index (rDRI) [27]. Myeloablative conditioning or reduced-intensity conditioning regimen was defined according to Giralt et al. [28]. Non-hematological adverse events were recorded in accordance with the National Cancer Institute Common Terminology Criteria for Adverse Events version 5.0.

### Exploratory studies

Peripheral blood serum was collected before the initiation of the conditioning regimen (pre-allo-HSCT) and on days 0, 14, and 28 after allo-HSCT (Fig. 1). The patients’ blood samples were centrifuged at 1500 g for 10 min at 4 °C (Kubota, Tabletop Cooling Centrifuge 2800, Tokyo, Japan). All oxidative stress marker levels were measured using the following enzyme-linked immunosorbent assay kits: HSP-70 (ADI-EKS-715; Enzo Life Sciences, Farmingdale, NY, USA), Trx-1 (ELH-TRDX1; RayBiotech Life, Inc. Peachtree Corners, GA, USA), asymmetric dimethylarginine (ADMA) (KR7828; Immundiagnostik AG, Bensheim, Germany), diacron-reactive oxygen metabolite (d-ROM) (DI-003b; Wismerll Co., Ltd., Tokyo, Japan), and nitric oxide (NO) (KGE001; R&D Systems, Inc., Minneapolis, MN, USA). Cytokine profiles were analyzed using Bio-Plex Pro™ Human Th17 Cytokine 15plex (171AA001M; Bio-Rad Laboratories, Inc., California, CA, USA). The kits were used according to the manufacturers’ instructions.

### Statistical analyses

Assuming cumulative incidence rates of severe aGVHD rates of 5% in the teprenone group and 20% in the control group, the required sample size would afford a statistical power of 52.8% with a two-sided 0.05 type 1 error of 40 patients per arm. However, owing to the entry deadline, recruitment was closed, and a total of 40 cases were included in this study. The cumulative incidence rate of severe aGVHD of 20% in the control group was based on previous reports [29–31]. A minimization method was used for randomization. The stratification factors were stem cell source (cord blood or not) and GVHD prophylaxis (PTCy or not) because of the lower cumulative incidence of aGVHD in allo-HSCT using these settings [32–34]. Central randomization was used for allocation concealment.

The cumulative incidence rates of severe aGVHD, relapse, and NRM in the groups were compared using Gray’s test. Relapse or NRM was considered a competing event, and relapse and death owing to non-GVHD causes were considered competing events for aGVHD and cGVHD. OS probabilities were assessed using the Kaplan–Meier method, and the groups were compared using the log-rank test. All statistical tests were two-sided, and *P* < 0.05 was considered statistically significant unless otherwise indicated. For oxidative stress marker and cytokine analyses, *P*-values calculated from the Wilcoxon matched-pairs signed-rank test were adjusted using the Benjamini–Hochberg method (i.e., adjusted *p*-value) and used to control the false discovery rate (FDR) [35]. Statistically significant differences between the two profiles were defined as *P* < FDR = 0.10. All statistical analyses were performed using EZR version 1.65 (Saitama Medical Center, Jichi Medical University, Japan) [36] and GraphPad Prism version 9 (GraphPad Software, San Diego, CA, USA).

## Results

### Patient characteristics

From May 2022 to February 2023, 40 patients were enrolled (21 in the control group and 19 in the teprenone group) (Fig. 1). The last follow-up was performed on April 30, 2024. Patient characteristics are summarized in Table 1. The patients’ baseline characteristics were well-balanced in terms of the median age, proportion of male sex, ECOG-PS ≤ 1, HCT-CI ≤ 2, previous history of allo-HSCT, complete remission at allo-HSCT, disease status at allo-HSCT, and rDRI. In contrast, the proportion of myeloid neoplasms was higher in the control group than in the teprenone group. Previous reports have identified numerous risk factors for aGVHD, including female donor-to-male recipient, cytomegalovirus (CMV) negativity in both donor and recipient, stem cell source, and conditioning regimen [31, 37, 38]. In the present study, the peri-allo-HSCT factors described above were similar in both groups. On the other hand, there was a higher proportion of patients in the control group with a myeloid neoplasms and previous history of allo-HSCT (All five patients had grade 1 aGVHD and/or mild cGVHD after first allo-HSCT and received second allo-HSCT after relapse). Of the 11 patients whose stem cell source was BM, 7 were MUD (control, *n* = 4; teprenone, *n* = 3) and 4 were 1-locus MMUD (control, *n* = 2; teprenone, *n* = 2). On the other hand, of the 18 patients whose stem cell source was PBSCs, 1 was MSD (control, *n* = 0; teprenone, *n* = 1), 5 were MUD (control, *n* = 2; teprenon, *n* = 3), and 12 were haploidentical sibling donor (control, *n* = 7; teprenone, *n* = 5). For GVHD prophylaxis, there was well-balanced with regard to GVHD prophylaxis between the two groups.

**Table 1:** Patient characteristics

		Total (40)	Control (21)	Teprenone (19)
Age (years), median (range)		57 (19–70)	60 (19–70)	56 (22–68)
Sex, *n* (%)	Male	23 (57.5)	11 (52.4)	12 (63.2)
	Female	17 (42.5)	10 (47.6)	7 (36.8)
ECOG-performance status^*^, *n* (%)	0–1	38 (95.0)	21 (100.0)	17 (89.5)
	2–4	2 (5.0)	0 (0.0)	2 (10.5)
HCT-CI^†^,* n* (%)	0–2	29 (72.5)	16 (76.2)	13 (68.4)
	≥ 3	11 (27.5)	5 (23.8)	6 (31.6)
Disease^‡^, *n* (%)	Myeloid neoplasms	24 (60.0)	16 (76.2)	8 (42.1)
	Lymphoid neoplasms	15 (37.5)	5 (23.8)	10 (52.6)
	MPAL	1 (2.5)	0 (0.0)	1 (5.3)
Previous history of allo-HSCT, *n* (%)	Yes	5 (12.5)	4 (19.0)	1 (5.3)
	1st allo-HSCT to relapse (days)	825 (195–1813)	539 (195–1813)	911 (NA–NA)
	Any type of GVHD after 1st allo-HSCT^§^	5	4	1
	No	35 (87.5)	17 (81.0)	18 (94.7)
Disease status at allo-HSCT, *n* (%)	CR	23 (57.5)	11 (52.4)	12 (63.2)
	non-CR or untreated	17 (42.5)	10 (47.6)	7 (36.8)
Refined disease risk index^||^, *n* (%)	Low/Intermediate	23 (57.5)	11 (52.4)	12 (63.2)
	High/Very high	17 (42.5)	10 (47.6)	7 (36.8)
Female to male, *n* (%)	Yes	5 (12.5)	2 (9.5)	3 (15.8)
	No	35 (87.5)	19 (90.5)	16 (84.2)
CMV status (donor/recipient: − / −), *n* (%)	Yes	2 (5.0)	1 (4.8)	1 (5.3)
	No	38 (95.0)	20 (95.2)	18 (94.7)
Stem cell source, *n* (%)	BM	11 (27.5)	6 (28.6)	5 (26.3)
	PBSCs	18 (45.0)	9 (42.9)	9 (47.4)
	CB	11 (27.5)	6 (28.6)	5 (26.3)
Conditioning regimen^¶^, *n* (%)	MAC	19 (47.5)	9 (42.9)	10 (52.6)
	RIC	21 (52.5)	12 (57.1)	9 (47.4)
GVHD prophylaxis, *n* (%)	TAC + MTX ± rATG	20 (50.0)	12 (57.1)	8 (42.1)
	TAC + MMF ± PTCY	20 (50.0)	9 (42.9)	11 (57.9)

^*^Graded according to the ECOG guidelines [25]. ^†^Defined according to the report by Sorror ML, et al. [26]. ^‡^Myeloid neoplasms were including acute myeloid leukemia (*n* = 13), myelodysplastic syndrome (MDS) (*n* = 7), myeloproliferative neoplasms (MPN) (*n* = 3) and MDS/MPN (*n* = 1), while lymphoid neoplasms were including acute lymphoblastic leukemia (*n* = 7), T-cell lymphoma (*n* = 7), and B-cell lymphoma (*n* = 1). ^§^All patients had Grade 1 aGVHD and/or mild cGVHD. ^||^Defined according to the report by Armand P, et al. [27]. ^¶^Defined according to the report by Giralt S, et al. [28] Abbreviations: *allo-HSCT* Allogeneic hematopoietic stem cell transplantation; *BM* Bone marrow; *CB* Cord blood; *CMV* Cytomegalovirus; *CR* Complete remission; *ECOG* Eastern Cooperative Oncology Group; *GVHD* Graft-versus-donor disease; *HCT-CI* Hematopoietic cell transplantation-specific comorbidity index; *MPAL* Mixed phenotype acute leukemia; ^*1*^* MAC* Myeloablative conditioning; *MMF* Mycophenolate mofetil; *MTX* Methotrexate; *PBSCs* Peripheral blood stem cells; *PTCY* Posttransplant cyclophosphamide; *rATG* Rabbit anti-thymocyte globulin; *RIC* Reduced intensity conditioning; *TAC* Tacrolimus

### Engraftment

All patients engrafted, and the median times for neutrophil and platelet engraftment in both groups were as follows: 16 (range, 13–22) vs. 15 (range, 11–23) days and 35 (range, 18–77) vs. 28 (range, 19–921) days, respectively. All patients had at least 95% donor chimerism in whole blood by day 30.

### Acute GVHD

**Fig. 2:**
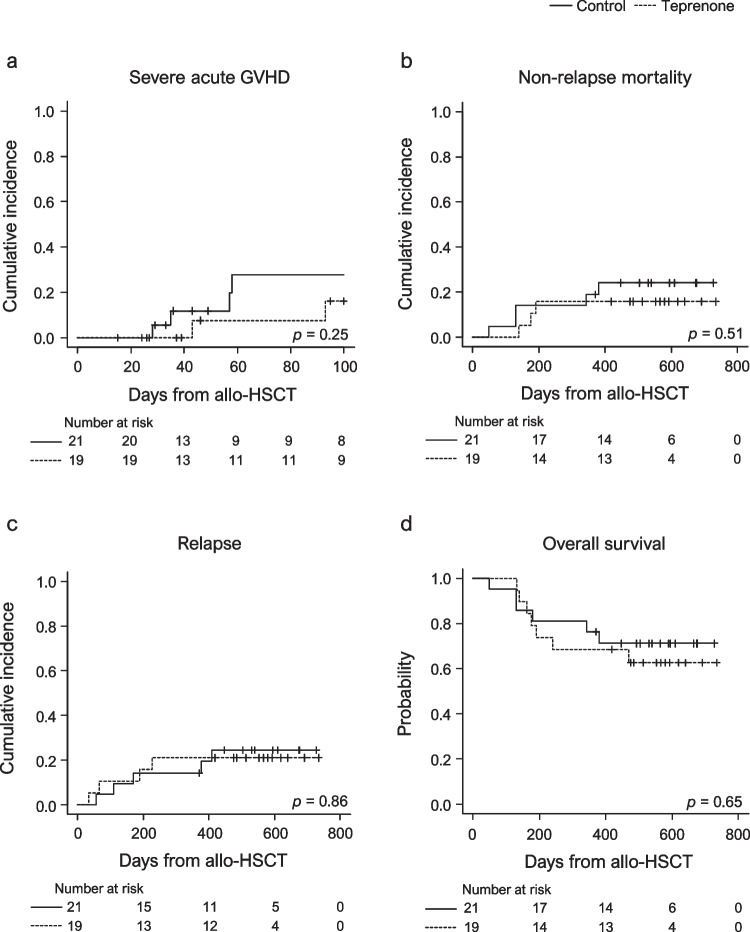
Patients’ clinical outcome with or without teprenone for graft-versus-host disease prevention. Cumulative incidence of (**a**) severe acute graft-versus-host disease, **b** non-relapse mortality, and (**c**) relapse and (**d**) overall survival in the study cohort. Abbreviations: allo-HSCT, allogeneic hematopoietic stem cell transplantation; GVHD, graft-versus-host disease

Mild aGVHD (grades I–II) occurred in 16 patients (control, *n* = 8; teprenone, *n* = 8). In contrast, nine patients had severe aGVHD (control, *n* = 6; teprenone, *n* = 3), with one patient in each group experiencing severe aGVHD beyond day 100 after allo-HSCT, so-called late-onset aGVHD [24]. The median times from allo-HSCT to the development of mild and severe aGVHD were 31 (range, 15–95) and 58 days (range, 28–112), respectively. Of the nine patients diagnosed with severe aGVHD, none were diagnosed with skin stage IV, one (11.1%) was diagnosed with liver stage II, and the remaining eight (88.9%) were diagnosed with lower gastrointestinal (GI) tract stage II [23]. There were no significant differences in the cumulative incidence of severe aGVHD by day 100 after allo-HSCT between the two groups (Fig. 2a, 27.9%; 95% confidence interval [CI], 7.7–53.0 vs. 16.1%; 95% CI, 2.3–41.4; *p* = 0.25).

### Toxicity, infectious complications, and non-relapse mortality

None of the 19 patients administered teprenone developed adverse events or discontinued teprenone. Bacterial infections complicated 12 patients (control, *n* = 7; teprenone, *n* = 5) by day 100 after allo-HSCT. Other infectious complications included CMV viremia (control, *n* = 4; teprenone, *n* = 0), BK virus cystitis (control, *n* = 4; teprenone, *n* = 1), and reactivation of human herpes virus 6 (control, *n* = 2; teprenone, *n* = 0). Five patients in the control group (disease progression, *n* = 1; GVHD, *n* = 2; transplant-associated thrombotic microangiopathy [TA-TMA], *n* = 1; pneumonia, *n* = 1) and six patients in the teprenone group (disease progression, *n* = 3; GVHD, *n* = 1; TA-TMA, *n* = 1; sinusoidal obstruction syndrome/veno-occlusive disease, *n* = 1) died within 1 year after allo-HSCT. NRM at 1 year after allo-HSCT was not significantly different between 0the two groups (Fig. 2b, 19.0%; 95% CI, 5.7–38.3 vs. 15.8%; 95% CI, 3.7–35.6; *p* = 0.51).

### Relapse, chronic GVHD, and survival

Each patient was followed up for at least 1 year after allo-HSCT unless dead, and the median follow-up period after allo-HSCT was comparable between the two groups (531 [range, 49–728 days] vs. 484 [range, 132–735] days). Five patients in the control group and four in the teprenone group relapsed at a median of 169 (range, 56–409) days and 127 (range, 33–227) days after allo-HSCT. Cumulative incidence of relapse (CIR) at 1 year after allo-HSCT was not significantly different in the two groups (Fig. 2c, 14.3%; 95% CI, 3.4–32.7 vs. 21.1%; 95% CI, 6.2–41.7; *p* = 0.86). cGVHD developed in 20 of the 36 patients who survived without relapse or died beyond day 100. Among these patients (control, *n* = 19; teprenone, *n* = 17), eight (control, *n* = 3; teprenone, *n* = 5) had mild cGVHD, whereas 12 (control, *n* = 7; teprenone, *n* = 5) had moderate-to-severe cGVHD. The cumulative incidence rate of moderate-to-severe cGVHD at 1 year after allo-HSCT was not significantly different between the groups (data not shown, 48.2%; 95% CI, 21.9–70.5 vs. 32.7%; 95% CI, 10.8–57.0; *p* = 0.56). Furthermore, the OS rates at day 100 and 1 year after allo-HSCT were not significantly different between the two groups (Fig. 2d) (95.2%; 95% CI, 71.0–99.0 vs. 100%; 95% CI, not estimated and 76.2%; 95% CI, 51.9–89.3 vs. 68.4%; 95% CI, 42.8–84.4; *p* = 0.65).

### Alternations of oxidative stress markers during allo-HSCT

**Fig. 3:**
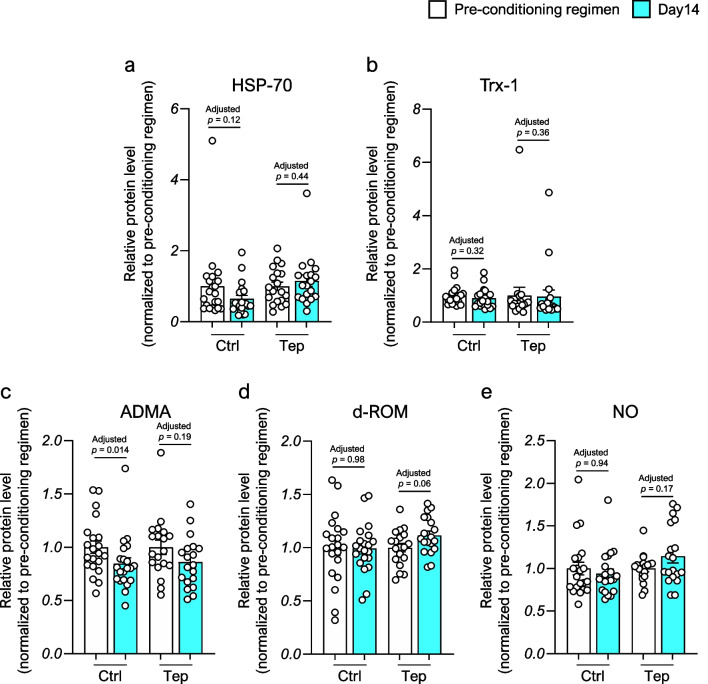
The alternation of oxidative stress marker levels in the serum before initiation of conditioning regimen and on day 14 after allo-hematopoietic stem cell transplantation between the control and the teprenone group. Data are presented as the mean ± SEM. Statistical significance was assessed using Wilcoxon matched-pairs signed rank test with Benjamini–Hochberg correction (**a–e**). Abbreviations: ADMA, asymmetric dimethylarginine; d-ROM, diacron-reactive oxygen metabolite; HSP-70, Heat Shock Protein-70; NO, nitric oxide; Tep, teprenone; Trx-1, thioredoxin-1

Next, we evaluated the alterations in HSP-70 and Trx-1, which are induced by GGA, as previously reported [12, 13], before and after oral teprenone therapy. A comparison of serum antioxidant enzyme levels from pre-allo-HSCT (baseline) to day14 after allo-HSCT revealed that HSP-70 levels were preserved on day 14 after allo-HSCT in the teprenone group, but the difference was not significant (Fig. 3a and b). Furthermore, we investigated other oxidative stress markers, including ADMA, whose pre-allo-HSCT levels are associated with NRM and OS [39]; d-ROM, an index of ROS products known to promote GVHD pathogenesis [40, 41]; and NO, whose pre-allo-HSCT levels are associated with NRM [42]. When these oxidative stress markers were compared, the ADMA level in the control group and the d-ROM level in the teprenone group fluctuated significantly on day 14 after allo-HSCT compared with baseline (Fig. 3c–e).

### Cytokine profiles during allo-HSCT

**Fig. 4:**
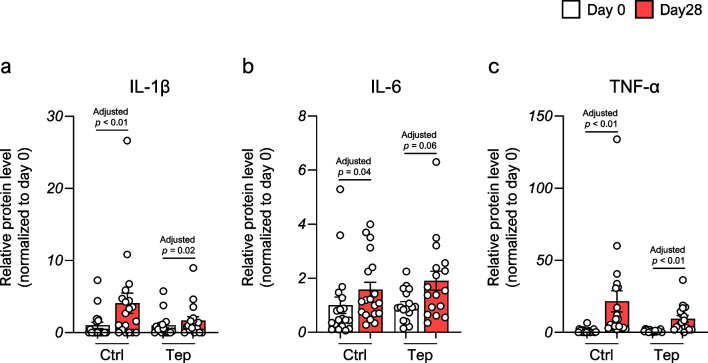
The alternation of interleukin (IL)−1β, IL-6, and tumor necrosis factor-α levels in the serum induced by allogeneic hematopoietic stem cell transplantation between the control and the teprenone group. Data are presented as the mean ± SEM. Statistical significance was assessed using Wilcoxon matched-pairs signed rank test with Benjamini–Hochberg correction (**a–c**). Abbreviations: Ctrl, control; IL-1β, interleukin-1β; IL-6, interleukin-6; Tep, teprenone; TNF-α, tumor necrosis factor-α

As it has been previously reported that IL-1β, IL-6, and TNF-α are suppressed by GGA, which is the main component of teprenone [18, 19], we analyzed cytokine profiles of the serum at days 0 (baseline) and 28 after allo-HSCT in both groups. All the levels of these cytokines in both groups were significantly higher 28 days post-allo-HSCT compared with baseline; however, the proportion of increase from baseline in IL-1β and TNF-α levels was suppressed in the teprenone group compared with the control group (Fig. 4a–c). A similar trend was observed in the analysis of patients who developed severe aGVHD (Fig. S1a–c).

**Fig. 5:**
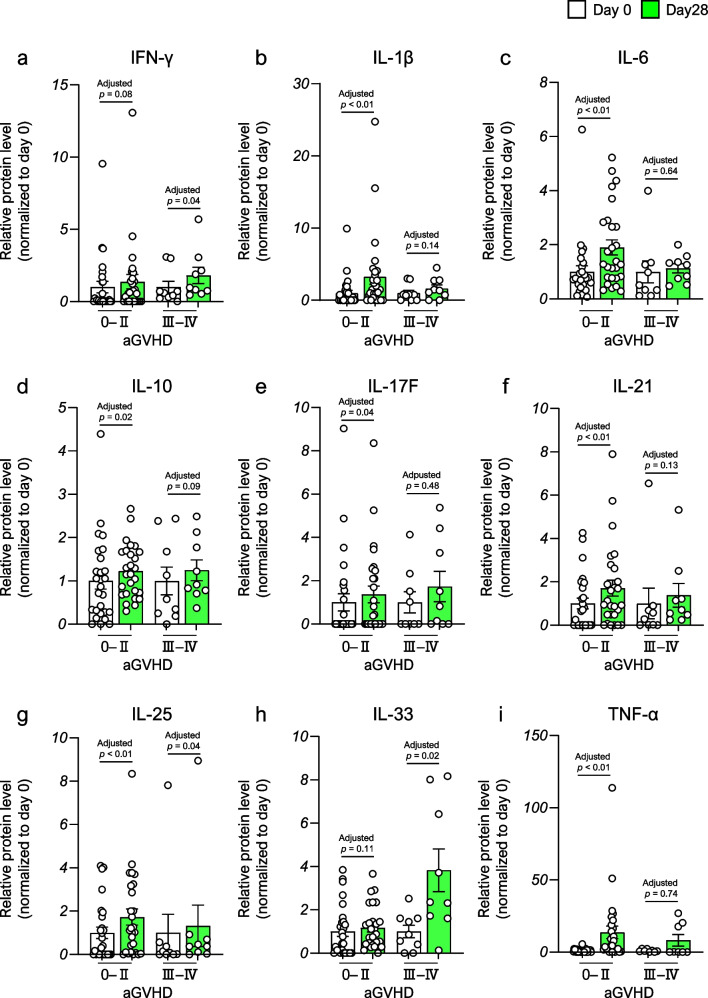
The alternation of proinflammatory cytokine levels in the serum induced by allogeneic hematopoietic stem cell transplantation between patients without and with severe acute graft-versus-host disease (grade 0–II vs. III–IV). Data are presented as the mean ± SEM. Statistical significance was assessed using Wilcoxon matched-pairs signed rank test with Benjamini–Hochberg correction (**a–i**). Abbreviations: aGVHD, acute graft-versus-host disease; IFN-γ, interferon-γ; IL-1β, interleukin-1β; IL-6, interleukin-6; IL-10, interleukin-10; IL-17F, interleukin-17F; IL-21, interleukin-21; IL-25, interleukin-25; IL-33, interleukin-33; TNF-α, tumor necrosis factor-α

Finally, to explore the effect of the alteration in allo-HSCT-induced cytokines from baseline on the subsequent development of severe aGVHD, we compared the cytokine profiles between patients without severe aGVHD and those with severe GVHD. All cytokine levels, except IL-33, were significantly increased in patients without severe aGVHD (Fig. 5a–i). As the development of aGVHD before day 28 following allo-HSCT may affect cytokine profiles at day 28 [20, 21], we divided this group into two groups, patients without aGVHD or with mild aGVHD beyond day 28 after allo-HSCT and those with mild aGVHD before day 28 after allo-HSCT, and performed the same analysis. The IL-1β, IL-10, IL-17F, IL-21, IL-25, and TNF-α levels in the former group while the IL-6 level in the latter group were significantly elevated from baseline (Fig. S2a–i). In contrast, significant increases in levels of IFN-γ, IL-10, IL-25, and IL-33 were found in patients with severe aGVHD (Fig. 5a, d, g, and h). Interestingly, IL-33 was the sole cytokine that was significantly elevated on day 28 after allo-HSCT compared with baseline only in patients with severe aGVHD.

## Discussion

This trial investigated whether the use of teprenone in combination with conventional GVHD prophylaxis in peri-allo-HSCT management contributes to a reduction in severe aGVHD via the induction of antioxidant enzymes. Unfortunately, we could not demonstrate an additional effect of teprenone on GVHD prophylaxis. However, teprenone was orally administered for 21 days from the initiation of the conditioning regimen with no effect on engraftment or apparent adverse events attributable to teprenone. In addition, no increase in bacterial and/or viral infections in the early post-allo-HSCT period compared with the control groups and similar 1-year NRM and OS in both groups indicated the feasibility and safety of teprenone in combination with conventional GVHD prophylaxis for peri-allo-HSCT management.

Immunomodulators such as abatasept, siltagliptin, and vedolizumab combined with conventional GVHD prophylaxis has been recently investigated to prevent the development of severe aGVHD after allo-HSCT, with favorable results [4–6]. We focused on oxidative stress, which is increased by conditioning regimens [10, 11]. Thus, we added teprenone, which induces the antioxidant enzyme Trx-1, which prevented GVHD in preclinical models while maintaining GVL effects, to conventional GVHD prophylaxis [12, 13, 17]. A potential reason for the lack of a clinical effect of teprenone in this study is as follows: Our exploratory study found that oral teprenone for 21 days from the initiation of the conditioning regimen did not significantly alter serum HSP-70 and Trx-1 on day14 after allo-HSCT compared with pre-allo-HSCT. In addtion, the increase from baseline in proinflammatory cytokines, such as IL-1β and TNF-α, at day 28 after allo-HSCT was tended to be suppressed in the teprenone group compared to the control group, but not sufficiently. Combined with the lack of difference in the cumulative incidence of severe aGVHD between the two groups, this result suggests that the teprenone dose administered in this study was for gastric mucosal protection, which may not be the optimal dose for GVHD prophylaxis in patients undergoing allo-HSCT. Sitagliptin contributed to the prevention of aGVHD while maintaining GVL effects in preclinical model; however, it only reduce the incidence of aGVHD when used at high dose in addition to conventional GVHD prophylaxis in clinical models [5, 43, 44]. Thus, increasing the dose of teprenone with attention to safety potentially lead to the proof of our hypothesis. Regarding GVL effects, considering the favorable balance in the patient baseline characteristics, including rDRI of the two groups, and the absence of significant differences in CIR at 1 year after allo-HSCT, it was suggested that at the dose of teprenone used in this study, short-term administration, at least during peri-allo-HSCT management, would not affect subsequent GVL effects.

Although advancements in prevention and supportive care have improved mortality rates after allo-HSCT, severe aGVHD still leads to a high risk of NRM [31, 45]. IL-33, a ligand for the suppression of tumorigenicity 2, which is one of the most effective markers for predicting clinical aGVHD [46, 47], has recently been highlighted in the context of GVHD. Reichenbach et al. reported significantly elevated levels of IL-33 protein produced by host non-hematopoietic cells of the GI tract in both mouse models and patients with aGVHD, compared with samples without aGVHD [48]. In addition, as a costimulatory signal, IL-33 has been suggested to play a crucial role in promoting the production of donor Th1 cells that infiltrate GVHD target tissues [49]. To the best of our knowledge, in the only report investigating serum IL-33 levels before and after allogeneic HSCT, IL-33 levels at day 28 were significantly elevated in patients with aGVHD compared with those without aGVHD in the training and validation cohorts. However, there were no data on alterations from day 0 (baseline) or on patients with severe aGVHD only [50]. Our finding of a significant increase in IL-33 levels on day 28 after allo-HSCT compared with day 0 in patients who subsequently developed severe aGVHD (8/9 cases involved severe GI tract aGVHD) suggests that IL-33 may be a useful biomarker for predicting the subsequent development of severe aGVHD of the GI tract. Further studies with larger patient cohorts are required to confirm our findings of the present study.

Our study has some limitations. First, this was an open-label, non-blinded study; thus, there is potential for bias. Second, this study included a small number of patients from a single institution, and the results are not definitive. Our study had a low power of 52.8% at a one-sided 0.05% significance level, but this setting number was that we thought would be achievable or realistic within the study period, estimated based on the number of allo-HSCT at our institution. However, due in part to the pandemic of COVID-19, only half of the planned number of patients were recruited as of the entry deadline, and our study was terminated. Third, in the patient background, the control group had a higher proportion of myeloid neoplasms and previous history of allo-HSCT. Allo-HSCT in patients with germline DDX41 variants has been suggested to be associated with the development of severe GVHD [51, 52]. However, the detection frequency in unselected acute myeloid leukemia patient cohorts is known to be very low (not evaluated in this study), thus we believe that the potential impact on this study was little. On the other hand, GVHD (any types) after first allo-HSCT and time between first allo-HSCT and relapse has been reported to be a risk factor for the development of severe aGVHD after the second allo-HSCT [53]. In our study, all five patients who received second allo-HSCT had mild aGVHD and/or cGVHD after the first allo-HSCT, and the time between the first allo-HSCT and relapse tended to be shorter in the control group. Therefore, these factors might influence the results. Finally, because this study was conducted in Japan and previous studies using registry data of cellular therapy have reported a lower risk of aGVHD after allo-HSCT among Japanese patients than among other races [54, 55], the application of these results to other countries warrants further investigation.

In conclusion, this trial could not demonstrate that teprenone significantly reduces severe aGVHD after allo-HSCT at the dose employed for gastric mucosal protection in Japan. However, our study suggests the feasibility and safety of adding teprenone to conventional GVHD prophylaxis in peri-allo-HSCT management. By exploring the dose that can induce antioxidant enzymes, teprenone may be an important component of the armamentarium for reducing the incidence of severe aGVHD after allo-HSCT.

## Supplementary information

Below is the link to the electronic supplementary material.

ESM 1 (DOCX 184 KB)

## Data Availability

No datasets were generated or analysed during the current study.
