# Supplementary material for: A randomized controlled trial of conventional GVHD prophylaxis with or without teprenone for the prevention of severe acute GVHD
Source: Ann Hematol. 2025 Feb 24;104(3):1917–29. doi: 10.1007/s00277-025-06269-2 (PMC12031882; doi:10.1007/s00277-025-06269-2)
Supplement: Supplementary file 1 [file 277_2025_6269_MOESM1_ESM.docx]

Figure S1

Fig. S1. The alternation of interleukin (IL)-1β, IL-6, and tumor necrosis factor-α levels in the serum induced by allogeneic hematopoietic stem cell transplantation between the control and the teprenone group in patients with severe acute graft-versus-host disease (grade Ⅲ–Ⅳ). Data are presented as the mean ± SEM. Statistical significance was assessed using Wilcoxon matched-pairs signed rank test with Benjamini–Hochberg correction (a–c). Abbreviations: Ctrl, control; IL-1β, interleukin-1β; IL-6, interleukin-6; Tep, teprenone; TNF-α, tumor necrosis factor-α

Figure S2

Fig. S2. The alternation of inflammatory cytokine levels in the serum induced by allogeneic hematopoietic stem cell transplantation (allo-HSCT) between patients without acute graft-versus-host disease (aGVHD) (grade 0) or with mild aGVHD (grade Ⅰ–Ⅱ) beyond day 28 after allo-HSCT and with mild aGVHD. Data are presented as the mean ± SEM. Statistical significance was assessed using Wilcoxon matched-pairs signed rank test with Benjamini–Hochberg correction (a–i). Abbreviations: aGVHD, acute graft-versus-host disease; allo-HSCT, allogeneic hematopoietic stem cell transplantation; IFN-γ, interferon-γ; IL-1β, interleukin-1β; IL-6, interleukin-6; IL-10, interleukin-10; IL-17F, interleukin-17F; IL-21, interleukin-21; IL-25, interleukin-25; IL-33, interleukin-33; TNF-α, tumor necrosis factor-α
